# CD4+ Memory T Cells at Home in the Tissue: Mechanisms for Health and Disease

**DOI:** 10.3389/fimmu.2018.02394

**Published:** 2018-10-16

**Authors:** David Schreiner, Carolyn G. King

**Affiliations:** Immune Cell Biology Lab, Department of Biomedicine, University Hospital Basel, University of Basel, Basel, Switzerland

**Keywords:** CD4 T cell memory, resident memory, infection, autoimmunity, vaccine

## Abstract

During the last 10 years, a population of clonally expanded T cells that take up permanent residence in non-lymphoid tissues has been identified. The localization of these tissue resident memory (TRM) cells allows them to rapidly respond at the site of antigen exposure, making them an attractive therapeutic target for various immune interventions. Although most studies have focused on understanding the biology underlying CD8 TRMs, CD4 T cells actually far outnumber CD8 T cells in barrier tissues such as lung and skin. Depending on the immune context, CD4 TRM can contribute to immune protection, pathology, or tissue remodeling. Although the ability of CD4 T cells to differentiate into heterogeneous effector and memory subsets has been well-established, how this heterogeneity manifests within the TRM compartment and within different tissues is just beginning to be elucidated. In this review we will discuss our current understanding of how CD4 TRMs are generated and maintained as well as a potential role for CD4 TRM plasticity in mediating the balance between beneficial and pathogenic immune responses.

## Introduction

Following activation, CD4+ T cells have the remarkable ability to differentiate into many different types of effectors. This diversity is required for the generation of effector T cells that are adapted to a particular immune context, as well as the development of long-lived and protective memory T cells. Compared to naïve T cells, memory CD4+ T cells are present at higher numbers, exhibit distinct trafficking behaviors, and generally have more rapid effector function following reinfection (1). Nevertheless, it is unclear how memory CD4 T cells positively versus negatively impact secondary immune responses. In the case of influenza vaccination, memory CD4 T cells have been shown to recognize conserved viral glycoproteins and may be able to provide cross-protective (heterotypic) protection to multiple influenza strains (2). This is in contrast to vaccine elicited antibodies which are directed against highly mutable viral proteins, resulting in the need for new vaccine formulations every year (3). On the other hand, memory CD4+ T cells are generally considered to be a barrier to transplantation tolerance and were recently reported to induce immune pathology in a mouse model of chronic viral infection (4, 5). The capacity of CD4 memory T cells to orchestrate divergent immune outcomes is in part related to their heterogeneity. Distinct types of effector T cells have been shown to give rise to apparently committed memory T cell lineages (6, 7). However, the stability and plasticity of these memory T cell subsets as well as their full impact on secondary immune responses are not yet understood.

**Figure 1:**
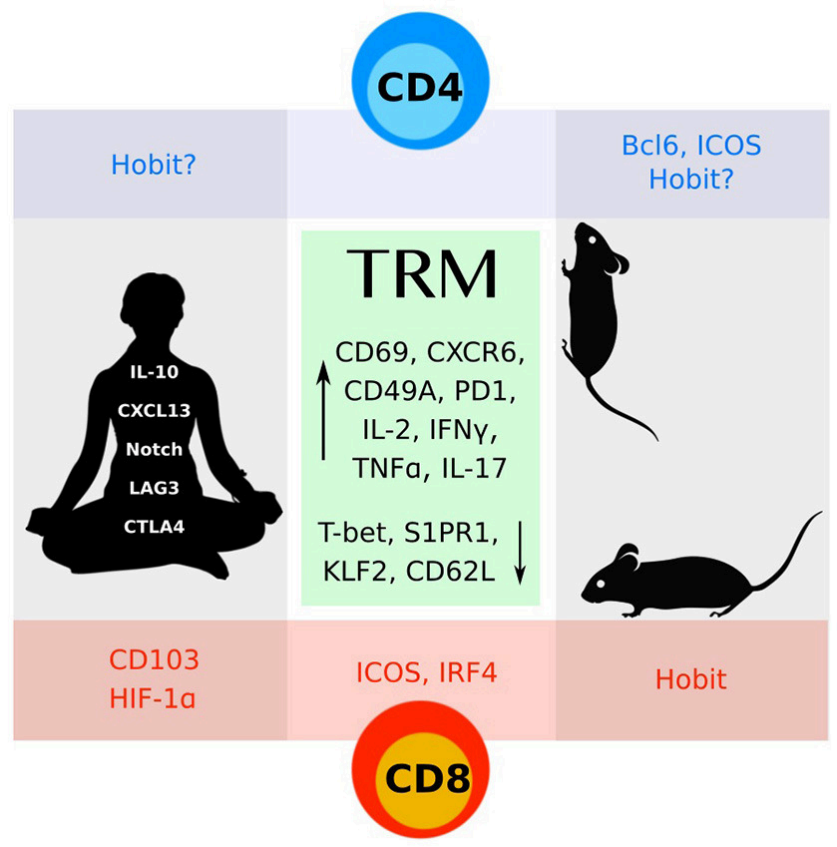
CD4 and CD8 TRM cells identified in mice and humans share many features (green box). More work remains to understand the distinct molecular programs that define these subsets.

More recently, an additional population of memory T cells localized within barrier tissues has been identified (8, 9). Due to their non-circulating status, these tissue resident memory (TRM) cells are uniquely poised to respond to antigen and execute immediate effector functions. While most studies have focused on understanding the cellular requirements and transcriptional basis of CD8 TRM differentiation, our understanding of CD4 TRM biology is less advanced. This review will highlight specific cellular and molecular requirements for CD4 TRM generation and survival within distinct organs, and in response to different pathogens or immune contexts. Since many of the phenotypic characteristics of CD4 TRM are shared with CD8 TRM cells, and are extensively reviewed elsewhere, we will focus our discussion on what sets CD4 TRM cells apart (Figure 1). Although much of this review focuses on findings generated in mouse models of infection or autoimmunity, we specifically highlight important observations made on human CD4 TRM throughout the manuscript.

The persistence of memory CD4 T cells in tissues has long been appreciated. In general, CD4 memory T cells appear to preferentially accumulate in mucosal tissues where they outnumber CD8 memory T cells (10, 11). Early work by the Jenkins lab showed that antigen and LPS led to CD4 T cell expansion and migration into tissues including lung, liver, gut, and salivary gland (12). Using whole mouse body imaging to quantify CD4 memory T cells, the authors demonstrated long-term survival of these cells in the tissue as well as their ability to rapidly produce IFNγ following recall activation. The residence status of CD4 TRM cells has been confirmed using parabiosis experiments (13). Similar to CD8 TRM cells, CD4 TRMs are protected from intravascular antibody staining and persist after treatment with FTY720, a sphingosine-1-phosphate receptor 1 (S1PR1) antagonist that prevents lymphocyte egress from lymphoid organs (14, 15). Phenotypically, CD4 TRM express constitutively high levels of PD1 and CD69 (13, 15, 16). CD69 is initially upregulated on activated T cells during priming by antigen presenting cells in the draining lymph node, after which it is downregulated, enabling S1PR1 mediated exit from lymphoid organs (17). T cells then enter the blood circulation en route to the site of inflammation, directed by upregulation of chemokines and adhesion markers as well as homing receptors that are imprinted during T cell interactions with antigen presenting cells in the lymph node (18, 19). Once in the tissue, TRM precursors begin to re-express CD69, although the signals mediating this upregulation are unclear. CD69 can be induced by signals through the T cell receptor (TCR), inflammatory cytokines such as IFNα, and oxygen availability; the independent contribution of these signals to CD4 TRM induction and maintenance is not yet known (20–22). A recent study demonstrated that human CD4 TRM cells isolated from distinct peripheral tissues express high levels of CD69 and are transcriptionally distinct from CD69–CD4 T cells (16). Human CD4 TRM cells share strong homology to mouse TRM cells, with increased expression of genes involved with TCR signaling, adhesion, and cytokines (23, 24).

## CD4 TRM generation: cytokine and antigen requirements

The common gamma-chain cytokines IL-2, IL-15, and IL-7 have well-established and fundamental roles in CD4 T cell biology (25). While IL-2 drives the initial expansion of activated CD4 T cell effectors, sustained IL-2 signals repress gene programs required for circulating/lymphoid CD4 memory T cell fate (26, 27). In contrast, IL-2 receptor mediated signals are essential for the generation of CD4 TRM cells. In both a Th1 model of viral pulmonary infection as well as a Th2 model of allergic asthma, the absence of IL-2R signaling on activated CD4 T cells resulted in their failure of these T cells to migrate into the lung and establish residence (28, 29). In agreement with these findings, work by the Swain group showed that late antigen recognition by CD4 T cells results in autocrine IL-2 production that supports the maintenance of CD4 TRM cells in the lung following influenza infection (30). An IL-2 independent pathway for CD4 TRM generation has also been identified in influenza infection (31). In this case, IL-15 was required during the priming phase of T cell activation, while late IL-15 signals were unnecessary for long-term CD4 TRM survival. This is in contrast to CD8 TRM cells in the skin and lung which depend on IL-15 for both their generation and maintenance (32). IL-7 signals are essential for the maintenance of both naïve and lymphoid homing CD4 memory cells (33). Lung TRM cells generated after influenza infection express higher levels of IL-7R compared to circulating effectors, and treatment with Fc-fused IL-7 can promote recruitment of circulating CD4 T cells into the lungs where they acquire a TRM-like phenotype and contribute to secondary immune responses (34). Consistent with this, IL-7R blockade in a Th2 allergy model led to decreased numbers of airway resident CD4 T cells (35). Further, in a skin model of contact hypersensitivity where IL-7 was specifically ablated in the skin, CD4 TRM cells failed to persist long term (36). These data indicate an important role for IL-7 signaling in either the recruitment or survival of CD4 TRM.

In addition to cytokines, antigen recognition by the TCR is required for CD4 T cell diversification into effector and memory subsets. The role of antigen in CD4 TRM maintenance, however, is less clear (37). Transfer of lung derived CD4 TRM cells into naïve recipients demonstrates the ability of these cells to home back to the lungs and survive in an antigen independent manner (13, 38). Consistent with this, CD4 TRM generated after influenza infection do not express Nur77 at late phases of infection, suggesting that they are no longer receiving TCR mediated signals (39). However, these latter data were generated using TCR transgenic OT-II cells which under some settings have been shown to undergo less heterogeneous differentiation compared to polyclonal CD4 T cells (40). Indeed, distinct CD4 T cell receptor clonotypes were recently described to be associated with distinct states of T cell activation following tuberculosis infection or within the tumor microenvironment, suggesting that access to antigen can regulate the extent of T cell heterogeneity (41, 42). It is interesting to note that Nur77 expression is reportedly increased in CD8 TRM cells and is required for long-term TRM survival in multiple tissues (43, 44). Although the survival of Nur77 deficient CD4 TRM cells has not yet been assessed, it is likely that depending on the tissue, intercellular interactions or infection context, CD4 TRM cells are differentially dependent on antigen and T cell receptor signaling.

## B cell requirements for CD4 TRM cells: relationship to TFH cells?

T cell interactions with B cells constitute another important aspect of CD4 TRM biology. In peripheral CD4 T cells, B cell interactions with T cells lead to upregulation of the transcription factor Bcl6, which in turn supports the differentiation of follicular helper (TFH) and memory T cells (45). TFH cells are a lymphoid resident population that share many phenotypic and molecular similarities with TRM cells, including high expression of CD69, PD1, ICOS, and P2X7R and a dependency on downregulation of KLF2 and S1PR1 for their induction (23, 46–49). Although these similarities suggest that B cells might be important for CD4 TRM generation, B cell deficiency led to enhanced Th2 TRM generation and maintenance in a house dust mite allergy model (28). Similarly, *Mycobacterium tuberculosis* (Mtb) infection resulted in the generation and maintenance of CD4 TRM in a B cell independent manner (38). In this case, however, CD4 TRM cell survival required T cell intrinsic expression of Bcl6 and ongoing signals through ICOS, both of which are also required to maintain TFH cells at late phases of immune responses in secondary lymphoid organs (50). The authors hypothesized that T cell interactions with ICOS-ligand expressing dendritic cells might be responsible for maintaining CD4 TRM cells. Highlighting the divergent role of B cells in CD4 TRM generation, another report showed that intranasal LCMV infection in the absence of B cells led to impaired Th1 TRM cell survival, despite enhanced initial recruitment of CD4 T cells to the lung (29). Although Bcl6 expression was not explicitly addressed in this model, it is interesting to note that in peripheral CD4 T cells, high levels of T-bet can impair the ability of Bcl6 to repress its target genes (51). Consistent with this idea, high levels of T-bet are associated with decreased generation of both CD4 and CD8 TRM (52, 53). Using a neonatal infection model, the Farber group showed that the susceptibility of infants to respiratory infections is a result of increased T-bet expression in effector T cells which impairs the ability of these cells to stabilize the TRM phenotype (52).

## TRM locations and intercellular interactions

CD4 TRM cells are often observed in cell clusters or ectopic lymphoid structures. The cellular content of these clusters can differ depending on the tissue and cytokine context. Several reports indicate that the presence or absence of these clusters can play a role in CD4 TRM mediated recall responses, protection from host pathology during chronic infection and tissue remodeling or repair during pathogen clearance. In this section we will overview the various tissues where CD4 TRM cells have been identified and discuss the potential of intercellular interactions to modulate local immunity.

### Skin

The skin is a barrier tissue home to a large proportion of the memory T cells in the body. Unlike CD8 TRM cells which localize in the epithelium, CD4 T cells are primarily found in the dermis where they demonstrate more motile behavior than their CD8 TRM counterparts (54). Using mice that express the photoconvertible molecule Kaede, a majority of CD4 T cells present in the skin were found to be in equilibrium with the circulation at steady state (55). CD69 expression on these CD4 T cells decreased as they trafficked to the draining lymph node, highlighting the infidelity of CD69 as a marker for CD4 tissue residency (55, 56). Following infection with herpes simplex virus or contact sensitization to induce local inflammation, IFNγ producing CD4 T cells increased in the skin and clustered around hair follicles in association with CCL5 producing CD11b and CD8 T cells (55). Depletion of CD8 T cells led to disruption of these clusters and impaired survival of skin CD4 TRM. The authors noted that the hair follicle is a rich site for chemokine and cytokine production as well as a major site of commensal colonization, both of which might play a role in facilitating the maintenance of immune cell clustering and reactivation of CD4 TRM cells. Skin CD4 TRM have also been identified following *Leishmania major* infection (57, 58). In this case, re-challenge at distal sites from the original infection results in rapid production of IFNγ and recruitment of inflammatory monocytes in a CXCR3 dependent manner. In addition to Th1 TRM cells, Th17 TRM cells have been identified in the skin following infection with *Candida albicans* (59). Although the primary IL-17 producing population in the skin at earlier time points is comprised of γδ T cells, CD4 αβ T cells recruited at later time points localize in the papillary dermis and upregulate expression of CD69 and CD103. CD103 is a relatively robust marker for CD8 TRM identification, but it is less uniformly expressed on CD4 TRM cells, and may represent a distinct subset that arises in a more limited number of tissues, possibly dependent on environmental TGFβ (60, 61). The skin is also home to resident regulatory T cells which may play a role in the pathogenesis of psoriasis, characterized by the development of TRM dependent skin lesions (62). In this case, CD4 regulatory TRM cells expand and produce low levels of IL-17. Here it is noteworthy that psoriasis can be treated with some success by IL-17 blockade, although the precise mechanism for this has not yet been resolved (63).

### Female reproductive tract

The female reproductive tract is a prime location for sexually transmitted as well as opportunistic infections, suggesting an important role for CD4 T cells in this tissue. Using a model of genital herpes, the Iwasaki group demonstrated the presence of CD4 TRM localized in clusters with CD11c+ MHC-II+ cells that are disconnected from the circulation (64). These CD4 TRM cells provide superior protection compared to circulating memory cells and are maintained by local interactions with macrophages. In this model, T cell stimulation by macrophages results in T cell production of IFNγ, leading to CCL5 expression by macrophages, thus creating a positive feedback loop for cell clustering. Protection upon re-challenge is also mediated by CD4 T cell production of IFNγ, which acts on stromal cells to prevent viral replication and spread. Although the role of antigen in this system is unclear, a prime and pull immunization strategy where antigen is administered subcutaneously followed by a one-time application of chemokines to the genital mucosa showed that local chemokines are sufficient to recruit but not maintain CD4 TRM (65). It was also reported that CD4 TRM may be reactivated by uninfected local dendritic cells and B cells that acquire antigen from infected epithelial cells (66). Neither dendritic cells nor B cells alone were required for CD4 TRM recall, but depletion of both populations resulted in loss of protection.

### Intestines

The intestinal mucosa is a unique barrier tissue that comes into contact with food and environmental antigens as well as commensals and infectious pathogens. Th17 TRM cells specific for segmented filamentous bacteria, a commensal microbe, have been identified in the lamina propria of mice (67). Human Th17 TRM cells that express the C-type lectin-like receptor CD161 have also been identified in the lamina propria of patients experiencing Crohn's disease (68). These cells can be activated to release IL-17 and IFNγ in response to inflammation induced IL-23, which further potentiates their colitogenic potential. Given that pathogenic and protective Th17 cells are regulated by the same environmental cytokines, it is likely that the distinct make-up of the microbiota plays a role in regulating heterogeneity within the gut CD4 TRM compartment. Commensals have also been shown to induce the formation of resident regulatory T cells that produce high amounts of TGFβ which promotes local tolerance (69, 70). In addition, a recent report shows an important contribution by TFH cells residing within Peyer's patches of the gut to maintaining intestinal health (71). Peyer's patches are lymphoid tissue comprised of immune cell sensors that are constantly exposed to luminal antigens and gut bacteria (72). Although the circulating status of these TFH cells has not been addressed, non-circulating TRM cells with high expression of CD69, similar to constitutive expression of CD69 on TFH cells, have been identified in other secondary lymphoid organs (73). Similar to CD8 TRM cells, TFH cells express high levels of the purinergic receptor P2X7R; ATP mediated signals through P2X7R are required to maintain the balance of commensals in this organ (48). In the absence of P2X7R signals, TFH cells expand, providing excessive help to germinal center B cells, ultimately resulting in excessive IgA production and dysregulation of local commensal populations. CD4 TRM cells in the gut can also be induced by oral infection with *Listeria monocytogenes* (74). Th1 TRMs generated in this model accumulate in the lamina propria and epithelium, and are maintained in an IL-15 independent manner. Th2 TRM cells can also be identified in the lamina propria and peritoneal cavity after infection with *Heligmosomoides polygyrus* (75). Re-challenge infection results in TCR dependent production of IL-4, IL-5, and IL-13, although TRM cells in the peritoneal cavity are additionally able to produce cytokines in response to IL-33 and IL-7 signals alone.

### Lungs

The lung is a highly vascularized barrier tissue in constant contact with a variety of airborne microbes and environmental pollutants. Infection or inflammation in the lung results in the formation of ectopic lymphoid tissue called inducible bronchus-associated lymphoid tissue (iBALT) (76). Similar to secondary lymphoid tissue, iBALT is characterized by compartmentalized B and T cell areas, follicular dendritic cells, antigen presenting cells, high endothelial venules, stromal cells and limited chemokine networks (77). It is likely that the cellular composition of iBALT plays a role in mediating the balance between protection and pathology in the lung, and may provide a niche for CD4 TRM cell survival.

Respiratory infections such as influenza induce the generation Th1 TRM cells that can be recalled to produce IFNγ and provide protection against heterotypic infections (60, 78). Th1 TRM cells express high levels of the integrins CD11a and VLA-1 (α1β1), the latter of which is required for Th1 TRM generation and survival following recall infection (13, 79). Two recent reports have described a transcriptional signature for Th1 TRM cells isolated from human lung (16, 61). Although Th1 TRM cells were sorted according to either CD69 or CD103 expression, both reports show a strong homology of Th1 TRM cells with CD8 TRM cells described in mice. In addition, CD4+CD103+ TRM cells express high levels of IL-21 receptor, TGFβ and genes associated with Notch signaling. After stimulation with anti-CD3/28 *in vitro*, these lung CD4 TRM demonstrated polyfunctional cytokine production, suggesting heterogeneity within this population. It should be noted, however, that as is normally the case with CD4 T cell cytokine production, ~50 percent of CD4 TRM cells did not produce any cytokines, suggesting heterogeneity in terms of CD4 T cell subset or activation state within this compartment. Th1 TRM cells are also generated after chronic infection with tuberculosis and are characterized by high expression of CXCR3 and low expression of KLRG1 (38, 80, 81). Adoptively transferred Th1 TRM cells migrate back to the lung parenchyma where they mediate superior Mtb control but produce less IFNγ compared to CD4 memory T cells in the vasculature. Both influenza and Mtb infection also induce the formation of sustained iBALT. In the case of influenza infection, the presence of iBALT is correlated with accelerated secondary CD4 T cell responses, suggesting that iBALT might provide a survival niche for Th1 TRM (76, 82). The presence of iBALT also plays a role in sustained neutralizing antibody production, indicating a role for ongoing interactions between CD4 TRM and B cells to support resident or memory B cell persistence. Given that the glycoproteins expressed on the surface of influenza are highly variable from year to year, the presence of neutralizing antibodies might not be expected to provide sufficient protection following recall infection. However, since CD4 and CD8 T cells typically recognize conserved epitopes from internal viral proteins, it is possible that prior exposure to influenza results in the accumulation of TRM cells capable of providing cross strain protection (2, 3). Here the maintenance of CD4 TRM cells within immune cell clusters and/or iBALT would provide a starting point for rapid renewal of secondary germinal center responses. Although the specifics of this scenario must still be addressed, a recent retrospective study found that the number of prior influenza exposures can be linked to demographic susceptibility to re-infection, and is associated with conserved antigen epitopes recognized by CD8 T cells (83).

In the case of chronic Mtb infection, iBALT induced by chronic Mtb infection is formed within the granuloma, which is essential for preventing pathogen dissemination (84). iBALT formation is associated with protective immune responses during latent tuberculosis in humans as well as macaque and mouse models of Mtb infection (85). A central component of iBALT in this model is the presence of CXCR5+ CD4 T cells which are initially recruited during the effector phase of infection. Although CXCR5 expression is required for T cell localization within the lung parenchyma and for the long-term persistence of these TRM cells, it is not yet clear how these TRM cells mediate protective immunity. One possibility is that CD4 TRM cells produce cytokines to recruit, organize, or maintain the local immune cell repertoire. If this is the case, it will be particularly important to examine how heterogeneity or division of labor within the CD4 TRM compartment might contribute to distinct aspects of local immunity. For example, do CXCR5+ CD4 TRM cells promote ongoing humoral immunity during Mtb infection? Although the role of antibodies in tuberculosis immunity has been controversial, recent work demonstrates distinct antibody qualities associated with latent vs. active infection (86). It will be interesting to determine whether CD4 T cell help to B cells plays a role in these observations. Another important question is how do CXCR5+ CD4 TRM cells relate to CXCR5- TRM cells or to CD4 TRM cells with the potential to produce IFNγ? One possibility is that CXCR5+ CD4 TRMs can be further recalled to differentiate into IFNγ effectors, similar to the differentiation potential of lymphoid CXCR5+ CD4 memory T cells (50, 87). In this case, CD4 TRMs would self-renew to maintain the presence of long-lived protective memory cells, while simultaneously differentiating into effectors to promote pathogen containment or clearance.

Aside from infections that induce type 1 interferon responses, excessive inflammatory responses in the lung can lead to pathogenic tissue remodeling such as observed in asthma, a chronic inflammatory lung disease triggered by sensitization to inhaled allergens (88). In both asthmatic patients as well as animal models of the disease, Th2 memory T cells generated during inflammatory outbreaks are thought to contribute to pathogenesis (28, 89, 90). Memory Th2 cells are maintained within iBALT, and are supported by Thy1+ IL7 producing lymphatic endothelial cells that express IL-33, CCL21, and CCL19 (91). In agreement with this, iBALT formation is also observed in patients with chronic obstructive pulmonary disease and excessive Th2 TRM responses associated with lung fibrosis (92, 93). Using a mouse model of allergy induced asthma, a recent report identified two distinct populations of tissue resident ST2+ Th2 T cells in the lung: one subset produces IL-5 to recruit eosinophils; the other produces amphiregulin, which programs the induction of inflammatory eosinophils ultimately leading to lung fibrosis (93). Amphiregulin production by regulatory TRM cells was also shown to prevent excessive host pathology following influenza infection, by inducing epithelium proliferation and repair after viral clearance (94). Here, the local production of pro-inflammatory cytokines IL-18 and IL-33 induced amphiregulin production by ST2+ Tregs, to promote tissue repair in a TCR independent manner. In these cases of both Th2 lung fibrosis and post influenza tissue repair, amphiregulin production by distinct TRM subsets promotes distinct types of local immunity. These findings highlight the importance of understanding how heterogeneity within the CD4 TRM compartment impacts the outcome of local immune responses.

## CD4 vs. CD8 TRM cells, mouse vs. human TRM cells: where do we stand?

As discussed above, most studies have focused on identifying phenotypic and molecular characteristics that discriminate CD8 TRM from circulating or lymphoid homing CD8 memory T cells. CD4 TRM studies have largely been placed within the context of these CD8 TRM findings, with the consensus being that the two cell types have much in common. A recent report by Farber and colleagues identified a core transcription signature shared by both CD4 and CD8 TRMs isolated from human organs (16). This signature, which was also largely shared with mouse TRM cells, included adhesion molecules such as CD103 and CD49a, chemokine receptors such as CXCR6 and CX3CR1, and genes known to be involved in dampening or inhibiting T cell responses, including PD1, DUSP6 and IL-10. On the other hand, CD4 TRM cells exhibited more clonal diversity compared to CD8 TRM, which likely reflects their underlying heterogeneity. In support of this, dimension reduction of RNAseq data revealed a broader spread of CD4 TRM cells compared to the tight grouping of CD8 TRM cells across the multiple tissues examined. These data are also consistent with a study that examined trafficking markers and cytokine production by human CD4 T cells distributed across several different lymphoid and non-lymphoid tissues at steady state (95). Using mass cytometry, the authors dissected CD4 TRM heterogeneity in terms of tissue specific expression of homing markers, along with the identification of distinct phenotypic and functional (in terms of cytokine production) CD4 T cell clusters within individual tissues. These studies underscore the complexity of CD4 TRM cell biology and lay the groundwork for applying more recently developed single cell technologies to the exploration of CD4 TRM cell heterogeneity.

Another important consideration is how closely the observations made in mouse models correlate with human TRM cell biology. It is important to note that a majority of mouse studies have examined antigen specific CD4 T cell responses, while most human studies have focused on a broader characterization of TRM cells. While many similarities exist, there are also some discrepancies, although whether these differences are a result of mouse-to-human comparison or CD4-to-CD8 comparison is unclear. For example, mouse CD8 TRM generated after LCMV infection express high levels of the transcription factor Hobit. Although Hobit could be identified in circulating human cytolytic CD4 T cells, it was not significantly upregulated in human TRM cells isolated from liver, gut, or skin (16). On the other hand, another study looking at CD103+ CD4 TRM cells isolated from human lung found increased mRNA expression of Hobit, although protein was not expressed (61). Importantly, in this latter study, CD103 was observed on ~10% of CD4 TRM cells isolated from the human lung, which is in contrast to mouse models either at steady state or after infection (61). Here the authors also identified a prevalent Notch signature in human CD4 TRM cells. Although the role of Notch signaling has not yet been addressed in mouse CD4 TRM cells, it was previously reported to be highly expressed in human CD8 TRM cells and is also required for the maintenance of mouse CD8 TRM cells (96, 97). Given the importance of Notch signaling in the survival of circulating memory CD4 T cells (98, 99), it seems likely that Notch would also play a role in CD4 TRM cells. Going forward, it will be important to connect observations in mouse models, which yield greater flexibility in terms of immune manipulation and organ harvest, with the valuable observations being made in human tissues. In addition, it will be essential to determine whether a minor subset of circulating TRM or pre-TRM cells can be identified, particularly after oral, intranasal or topical immunization leading to TRM induction. The identification of such cells would greatly aid the comparison of mouse and human TRM studies.

## Concluding remarks

CD4 TRM cells localized within barrier tissues are poised to provide immediate protection from re-challenge infection. In some cases, however, the long-term survival of CD4 TRM cells within inflammatory or autoimmune contexts can lead to host immunopathology. Understanding the cellular requirements and transcriptional basis underlying the acquisition and maintenance of CD4 TRM phenotype, function and heterogeneity is crucial for identifying ways in which CD4 TRM cells could be targeted for human health. One impediment to the detailed characterization of CD4 TRM cells is that the processing steps for isolation of TRM cells result in extremely poor cell recovery and the potential to bias against certain cell subsets (100). However, recent advances in multiplexed single cell imaging and single cell RNA transcriptomics in combination with TCR repertoire analysis will greatly assist in dissecting the relationship of CD4 TRM heterogeneity to cell activation state, function, and intercellular interactions (42, 101). It will additionally be important to examine how CD4 TRM cells respond to re-stimulation, whether they can self-renew, and whether or not they exhibit fate plasticity. Along these lines, secondary effector CD4 T cells responding to influenza in the lung contain both Th17 and TFH subsets, neither of which are present during primary infection (102). It will be of great value to determine whether such cells arise from distinct CD4 TRM precursors or whether they are newly generated in lymphoid organs. Understanding CD4 TRM flexibility during chronic infection or within the tumor microenvironment will also be important for assessing the potential of vaccines to target these populations.

## Author contributions

All authors listed have made a substantial, direct and intellectual contribution to the work, and approved it for publication.

### Conflict of interest statement

The authors declare that the research was conducted in the absence of any commercial or financial relationships that could be construed as a potential conflict of interest.

## References

[1] Chang JT Wherry EJ Goldrath AW. Molecular regulation of effector and memory T cell differentiation. Nat Immunol. (2014) 15:1104–15. 10.1038/ni.3031 25396352 PMC4386685

[2] Zens KD Chen JK Farber DL. Vaccine-generated lung tissue-resident memory T cells provide heterosubtypic protection to influenza infection. JCI Insight (2016) 1:e85832. 10.1172/jci.insight.85832 27468427 PMC4959801

[3] Devarajan P Bautista B Vong AM McKinstry KK Strutt TM Swain SL. New insights into the generation of CD4 memory may shape future vaccine strategies for influenza. Front Immunol. (2016) 7:136. 10.3389/fimmu.2016.00136 27148257 PMC4827017

[4] Espinosa JR Samy KP Kirk AD. Memory T cells in organ transplantation: progress and challenges. Nat Rev Nephrol. (2016) 12:339–47. 10.1038/nrneph.2016.9 26923209 PMC5341793

[5] Penaloza-MacMaster P Barber DL Wherry EJ Provine NM Teigler JE Parenteau L. Vaccine-elicited CD4 T cells induce immunopathology after chronic LCMV infection. Science (2015) 347:278–82. 10.1126/science.aaa2148 25593185 PMC4382081

[6] Hale JS Youngblood B Latner DR Mohammed AU Ye L Akondy RS. Distinct memory CD4(+) T cells with commitment to T follicular helper- and T helper 1-cell lineages are generated after acute viral infection. Immunity (2013) 38:805–17. 10.1016/j.immuni.2013.02.020 23583644 PMC3741679

[7] Keck S Schmaler M Ganter S Wyss L Oberle S Huseby ES. Antigen affinity and antigen dose exert distinct influences on CD4 T-cell differentiation. Proc Natl Acad Sci USA. (2014) 111:14852–7. 10.1073/pnas.1403271111 25267612 PMC4205596

[8] Schenkel JM Masopust D. Tissue-resident memory T cells. Immunity (2014) 41:886–97. 10.1016/j.immuni.2014.12.007 25526304 PMC4276131

[9] Thome JJ Yudanin N Ohmura Y Kubota M Grinshpun B Sathaliyawala T. Spatial map of human T cell compartmentalization and maintenance over decades of life. Cell (2014) 159:814–28. 10.1016/j.cell.2014.10.026 25417158 PMC4243051

[10] Yang L Yu Y Kalwani M Tseng TW Baltimore D. Homeostatic cytokines orchestrate the segregation of CD4 and CD8 memory T-cell reservoirs in mice. Blood (2011) 118:3039–50. 10.1182/blood-2011-04-349746 21791416 PMC3175781

[11] Sathaliyawala T Kubota M Yudanin N Turner D Camp P Thome JJ. Distribution and compartmentalization of human circulating and tissue-resident memory T cell subsets. Immunity (2013) 38:187–97. 10.1016/j.immuni.2012.09.020 23260195 PMC3557604

[12] Reinhardt RL Bullard DC Weaver CT Jenkins MK Preferential accumulation of antigen-specific effector CD4 T cells at an antigen injection site involves CD62E-dependent migration but not local proliferation. J Exp Med. (2003) 197:751–62. 10.1084/jem.20021690 12629067 PMC2193845

[13] Teijaro JR Turner D Pham Q Wherry EJ Lefrancois L Farber DL. Cutting edge: Tissue-retentive lung memory CD4 T cells mediate optimal protection to respiratory virus infection. J Immunol. (2011) 187:5510–4. 10.4049/jimmunol.1102243 22058417 PMC3221837

[14] Anderson KG Mayer-Barber K Sung H Beura L James BR Taylor JJ. Intravascular staining for discrimination of vascular and tissue leukocytes. Nat Protoc. (2014) 9:209–22. 10.1038/nprot.2014.005 24385150 PMC4428344

[15] Takamura S Yagi H Hakata Y Motozono C McMaster SR Masumoto T. Specific niches for lung-resident memory CD8+ T cells at the site of tissue regeneration enable CD69-independent maintenance. J Exp Med. (2016) 213:3057–73. 10.1084/jem.20160938 27815325 PMC5154946

[16] Kumar BV Ma W Miron M Granot T Guyer RS Carpenter DJ. Human tissue-resident memory T cells are defined by core transcriptional and functional signatures in lymphoid and mucosal sites. Cell Rep. (2017) 20:2921–34. 10.1016/j.celrep.2017.08.078 28930685 PMC5646692

[17] Cyster JG Schwab SR. Sphingosine-1-phosphate and lymphocyte egress from lymphoid organs. Annu Rev Immunol. (2012) 30:69–94. 10.1146/annurev-immunol-020711-075011 22149932

[18] Mikhak Z Strassner JP Luster AD. Lung dendritic cells imprint T cell lung homing and promote lung immunity through the chemokine receptor CCR4. J Exp Med. (2013) 210:1855–69. 10.1084/jem.20130091 23960189 PMC3754856

[19] Mora JR. Homing imprinting and immunomodulation in the gut: role of dendritic cells and retinoids. Inflamm Bowel Dis. (2008) 14:275–89. 10.1002/ibd.20280 17924560

[20] Shiow LR Rosen DB Brdickova N Xu Y An J Lanier LL. CD69 acts downstream of interferon-alpha/beta to inhibit S1P1 and lymphocyte egress from lymphoid organs. Nature (2006) 440:540–4. 10.1038/nature04606 16525420

[21] Labiano S Melendez-Rodriguez F Palazon A Teijeira A Garasa S Etxeberria I. CD69 is a direct HIF-1alpha target gene in hypoxia as a mechanism enhancing expression on tumor-infiltrating T lymphocytes. Oncoimmunology (2017) 6:e1283468. 10.1080/2162402X.2017.1283468 28507790 PMC5414881

[22] Testi R Phillips JH Lanier LL. Leu 23 induction as an early marker of functional CD3/T cell antigen receptor triggering. Requirement for receptor cross-linking, prolonged elevation of intracellular [Ca++] and stimulation of protein kinase C. J Immunol. (1989) 142:1854–60. 2466079

[23] Skon CN Lee JY Anderson KG Masopust D Hogquist KA Jameson SC. Transcriptional downregulation of S1pr1 is required for the establishment of resident memory CD8+ T cells. Nat Immunol. (2013) 14:1285–93. 10.1038/ni.2745 24162775 PMC3844557

[24] Mackay LK Minnich M Kragten NA Liao Y Nota B Seillet C. Hobit and Blimp1 instruct a universal transcriptional program of tissue residency in lymphocytes. Science (2016) 352:459–63. 10.1126/science.aad2035 27102484

[25] Rochman Y Spolski R Leonard WJ. New insights into the regulation of T cells by gamma(c) family cytokines. Nat Rev Immunol. (2009) 9:480–90. 10.1038/nri2580 19543225 PMC2814538

[26] Kalia V Sarkar S Subramaniam S Haining WN Smith KA Ahmed R. Prolonged interleukin-2Ralpha expression on virus-specific CD8+ T cells favors terminal-effector differentiation *in vivo*. Immunity (2010) 32:91–103. 10.1016/j.immuni.2009.11.010 20096608

[27] Pipkin ME Sacks JA Cruz-Guilloty F Lichtenheld MG Bevan MJ Rao A. Interleukin-2 and inflammation induce distinct transcriptional programs that promote the differentiation of effector cytolytic T cells. Immunity (2010) 32:79–90. 10.1016/j.immuni.2009.11.012 20096607 PMC2906224

[28] Hondowicz BD An D Schenkel JM Kim KS Steach HR Krishnamurty AT. Interleukin-2-dependent allergen-specific tissue-resident memory cells drive asthma. Immunity (2016) 44:155–66. 10.1016/j.immuni.2015.11.004 26750312 PMC4720536

[29] Hondowicz BD Kim KS Ruterbusch MJ Keitany GJ Pepper M. IL-2 is required for the generation of viral-specific CD4(+) Th1 tissue-resident memory cells and B cells are essential for maintenance in the lung. Eur J Immunol. (2018) 48:80–6. 10.1002/eji.201746928 28948612 PMC6215361

[30] McKinstry KK Strutt TM Bautista B Zhang W Kuang Y Cooper AM. Effector CD4 T-cell transition to memory requires late cognate interactions that induce autocrine IL-2. Nat Commun. (2014) 5:5377. 10.1038/ncomms6377 25369785 PMC4223689

[31] Strutt TM Dhume K Finn CM Hwang JH Castonguay C Swain SL. IL-15 supports the generation of protective lung-resident memory CD4 T cells. Mucosal Immunol. (2018) 11:668–80. 10.1038/mi.2017.101 29186108 PMC5975122

[32] Mackay LK Rahimpour A Ma JZ Collins N Stock AT Hafon ML. The developmental pathway for CD103(+)CD8+ tissue-resident memory T cells of skin. Nat Immunol. (2013) 14:1294–301. 10.1038/ni.2744 24162776

[33] Raeber ME Zurbuchen Y Impellizzieri D Boyman O. The role of cytokines in T-cell memory in health and disease. Immunol Rev. (2018) 283:176–93. 10.1111/imr.12644 29664568

[34] Kang MC Choi DH Choi YW Park SJ Namkoong H Park KS. Intranasal introduction of Fc-fused interleukin-7 provides long-lasting prophylaxis against lethal influenza virus infection. J Virol. (2015) 90:2273–84. 10.1128/JVI.02768-15 26656713 PMC4810684

[35] Yeon SM Halim L Chandele A Perry CJ Kim SH Kim SU. IL-7 plays a critical role for the homeostasis of allergen-specific memory CD4 T cells in the lung and airways. Sci Rep. (2017) 7:11155. 10.1038/s41598-017-11492-7 28894184 PMC5593957

[36] Adachi T Kobayashi T Sugihara E Yamada T Ikuta K Pittaluga S. Hair follicle-derived IL-7 and IL-15 mediate skin-resident memory T cell homeostasis and lymphoma. Nat Med. (2015) 21:1272–9. 10.1038/nm.3962 26479922 PMC4636445

[37] Thom JT Weber TC Walton SM Torti N Oxenius A. The salivary gland acts as a sink for tissue-resident memory CD8(+) T cells, facilitating protection from local cytomegalovirus infection. Cell Rep. (2015) 13:1125–36. 10.1016/j.celrep.2015.09.082 26526997

[38] Moguche AO Shafiani S Clemons C Larson RP Dinh C Higdon LE. ICOS and Bcl6-dependent pathways maintain a CD4 T cell population with memory-like properties during tuberculosis. J Exp Med. (2015) 212:715–28. 10.1084/jem.20141518 25918344 PMC4419347

[39] Bautista BL Devarajan P McKinstry KK Strutt TM Vong AM Jones MC Short-lived antigen recognition but not viral infection at a defined checkpoint programs effector CD4 T cells to become protective memory. J Immunol. (2016) 197:3936–49. 10.4049/jimmunol.1600838 27798159 PMC5113829

[40] Tubo NJ Pagan AJ Taylor JJ Nelson RW Linehan JL Ertelt JM. Single naive CD4(+) T cell*s* from a diverse repertoire produce different effector cell types during infection. Cell (2013) 153:785–96. 10.1016/j.cell.2013.04.007 23663778 PMC3766899

[41] Moguche AO Musvosvi M Penn-Nicholson A Plumlee CR Mearns H Geldenhuys H. Antigen availability shapes T cell differentiation and function during tuberculosis. Cell Host Microbe (2017) 21:695–706 e5. 10.1016/j.chom.2017.05.012 28618268 PMC5533182

[42] Azizi E Carr AJ Plitas G Cornish AE Konopacki C Prabhakaran S. Single-cell map of diverse immune phenotypes in the breast tumor microenvironment. Cell (2018) 174:1293–1308.e36. 10.1016/j.cell.2018.05.060 29961579 PMC6348010

[43] Boddupalli CS Nair S Gray SM Nowyhed HN Verma R Gibson JA. ABC transporters and NR4A1 identify a quiescent subset of tissue-resident memory T cells. J Clin Invest. (2016) 126:3905–16. 10.1172/JCI85329 27617863 PMC5096804

[44] Wang D Diao H Getzler AJ Rogal W Frederick MA Milner J. The transcription factor Runx3 establishes chromatin accessibility of cis-regulatory landscapes that drive memory cytotoxic T lymphocyte formation. Immunity (2018) 48: 659–74 e6. 10.1016/j.immuni.2018.03.028 29669249 PMC6750808

[45] Crotty S. Follicular helper CD4 T cells (TFH). Annu Rev Immunol. (2011) 29:621–63. 10.1146/annurev-immunol-031210-101400 21314428

[46] Fazilleau N McHeyzer-Williams LJ Rosen H McHeyzer-Williams MG. The function of follicular helper T cells is regulated by the strength of T cell antigen receptor binding. Nat Immunol. (2009) 10:375–84. 10.1038/ni.1704 19252493 PMC2712297

[47] Choi YS Kageyama R Eto D Escobar TC Johnston RJ Monticelli L. ICOS receptor instructs T follicular helper cell versus effector cell differentiation via induction of the transcriptional repressor Bcl6. Immunity (2011) 34:932–46. 10.1016/j.immuni.2011.03.023 21636296 PMC3124577

[48] Iyer SS Latner DR Zilliox MJ McCausland M Akondy RS Penaloza-Macmaster P. Identification of novel markers for mouse CD4(+) T follicular helper cells. Eur J Immunol. (2013) 43:3219–32. 10.1002/eji.201343469 24030473 PMC3947211

[49] Lee JY Skon CN Lee YJ Oh S Taylor JJ Malhotra D. The transcription factor KLF2 restrains CD4(+) T follicular helper cell differentiation. Immunity (2015) 42:252–64. 10.1016/j.immuni.2015.01.013 25692701 PMC4409658

[50] Pepper M Pagan AJ Igyarto BZ Taylor JJ Jenkins MK. Opposing signals from the Bcl6 transcription factor and the interleukin-2 receptor generate T helper 1 central and effector memory cells. Immunity (2011) 35:583–95. 10.1016/j.immuni.2011.09.009 22018468 PMC3208313

[51] Oestreich KJ Mohn SE Weinmann AS. Molecular mechanisms that control the expression and activity of Bcl-6 in TH1 cells to regulate flexibility with a TFH-like gene profile. Nat Immunol. (2012) 13:405–11. 10.1038/ni.2242 22406686 PMC3561768

[52] Zens KD Chen JK Guyer RS Wu FL Cvetkovski F Miron M. Reduced generation of lung tissue-resident memory T cells during infancy. J Exp Med. (2017) 214:2915–32. 10.1084/jem.20170521 28855242 PMC5626403

[53] Laidlaw BJ Zhang N Marshall HD Staron MM Guan T Hu Y. CD4+ T cell help guides formation of CD103+ lung-resident memory CD8+ T cells during influenza viral infection. Immunity (2014) 41:633–45. 10.1016/j.immuni.2014.09.007 25308332 PMC4324721

[54] Gebhardt T Whitney PG Zaid A Mackay LK Brooks AG Heath WR. Different patterns of peripheral migration by memory CD4+ and CD8+ T cells. Nature (2011) 477:216–9. 10.1038/nature10339 21841802

[55] Collins N Jiang X Zaid A Macleod BL Li J Park CO. Skin CD4(+) memory T cells exhibit combined cluster-mediated retention and equilibration with the circulation. Nat Commun. (2016) 7:11514. 10.1038/ncomms11514 27160938 PMC4866325

[56] Bromley SK Yan S Tomura M Kanagawa O Luster AD. Recirculating memory T cells are a unique subset of CD4+ T cells with a distinct phenotype and migratory pattern. J Immunol. (2013) 190:970–6. 10.4049/jimmunol.1202805 23255361 PMC3618989

[57] Glennie ND Volk SW Scott P. Skin-resident CD4+ T cells protect against Leishmania major by recruiting and activating inflammatory monocytes. PLoS Pathog. (2017) 13:e1006349. 10.1371/journal.ppat.1006349 28419151 PMC5409171

[58] Glennie ND Yeramilli VA Beiting DP Volk SW Weaver CT Scott P. Skin-resident memory CD4+ T cells enhance protection against Leishmania major infection. J Exp Med. (2015) 212:1405–14. 10.1084/jem.20142101 26216123 PMC4548053

[59] Park CO Fu X Jiang X Pan Y Teague JE Collins N. Staged development of long-lived T-cell receptor alphabeta TH17 resident memory T-cell population to Candida albicans after skin infection J Allergy Clin Immunol. (2017) 142:647–62. 10.1016/j.jaci.2017.09.042 29128674 PMC5943196

[60] Turner DL Farber DL. Mucosal resident memory CD4 T cells in protection and immunopathology. Front Immunol. (2014) 5:331. 10.3389/fimmu.2014.00331 25071787 PMC4094908

[61] Oja AE Piet B Helbig C Stark R van der Zwan D Blaauwgeers H. Trigger-happy resident memory CD4(+) T cells inhabit the human lungs. Mucosal Immunol. (2018) 11:654–67. 10.1038/mi.2017.94 29139478

[62] Sanchez Rodriguez R Pauli ML Neuhaus IM Yu SS Arron ST Harris HW Memory regulatory T cells reside in human skin. J Clin Invest. (2014) 124:1027–36. 10.1172/JCI72932 24509084 PMC3934172

[63] Hueber W Patel DD Dryja T Wright AM Koroleva I Bruin G. Effects of AIN457, a fully human antibody to interleukin-17A, on psoriasis, rheumatoid arthritis, and uveitis. Sci Transl Med. (2010) 2:52ra72. 10.1126/scitranslmed.3001107 20926833

[64] Iijima N Iwasaki A. T cell memory. A local macrophage chemokine network sustains protective tissue-resident memory CD4 T cells. Science (2014) 346:93–8. 10.1126/science.1257530 25170048 PMC4254703

[65] Shin H Iwasaki A. A vaccine strategy that protects against genital herpes by establishing local memory T cells. Nature (2012) 491:463–7. 10.1038/nature11522 23075848 PMC3499630

[66] Iijima N Linehan MM Zamora M Butkus D Dunn R Kehry MR. Dendritic cells and B cells maximize mucosal Th1 memory response to herpes simplex virus. J Exp Med. (2008) 205:3041–52. 10.1084/jem.20082039 19047439 PMC2605233

[67] Ivanov II Atarashi K Manel N Brodie EL Shima T Karaoz U. Induction of intestinal Th17 cells by segmented filamentous bacteria. Cell (2009) 139:485–98. 10.1016/j.cell.2009.09.033 19836068 PMC2796826

[68] Kleinschek MA Boniface K Sadekova S Grein J Murphy EE Turner SP. Circulating and gut-resident human Th17 cells express CD161 and promote intestinal inflammation. J Exp Med. (2009) 206:525–34. 10.1084/jem.20081712 19273624 PMC2699125

[69] Round JL Mazmanian SK. Inducible Foxp3+ regulatory T-cell development by a commensal bacterium of the intestinal microbiota. Proc Natl Acad Sci USA. (2010) 107:12204–9. 10.1073/pnas.0909122107 20566854 PMC2901479

[70] Atarashi K Tanoue T Shima T Imaoka A Kuwahara T Momose Y. Induction of colonic regulatory T cells by indigenous Clostridium species. Science (2011) 331:337–41. 10.1126/science.1198469 21205640 PMC3969237

[71] Proietti M Cornacchione V Rezzonico Jost T Romagnani A Faliti CE Perruzza L. ATP-gated ionotropic P2X7 receptor controls follicular T helper cell numbers in Peyer's patches to promote host-microbiota mutualism. Immunity (2014) 41:789–801. 10.1016/j.immuni.2014.10.010 25464855

[72] Reboldi A Arnon TI Rodda LB Atakilit A Sheppard D Cyster JG. IgA production requires B cell interaction with subepithelial dendritic cells in Peyer's patches. Science (2016) 352:aaf4822. 10.1126/science.aaf4822 27174992 PMC4890166

[73] Schenkel JM Fraser KA Masopust D. Cutting edge: resident memory CD8 T cells occupy frontline niches in secondary lymphoid organs. J Immunol. (2014) 192:2961–4. 10.4049/jimmunol.1400003 24600038 PMC3965619

[74] Romagnoli PA Fu HH Qiu Z Khairallah C Pham QM Puddington L. Differentiation of distinct long-lived memory CD4 T cells in intestinal tissues after oral *Listeria monocytogenes* infection. Mucosal Immunol. (2017) 10:520–30. 10.1038/mi.2016.66 27461178 PMC5272904

[75] Steinfelder S Rausch S Michael D Kuhl AA Hartmann S. Intestinal helminth infection induces highly functional resident memory CD4(+) T cells in mice. Eur J Immunol. (2017) 47:353–63. 10.1002/eji.201646575 27861815

[76] Hwang JY Randall TD Silva-Sanchez A. Inducible bronchus-associated lymphoid tissue: taming inflammation in the lung. Front Immunol. (2016) 7:258. 10.3389/fimmu.2016.00258 27446088 PMC4928648

[77] Randall TD. Bronchus-associated lymphoid tissue (BALT) structure and function. Adv Immunol. (2010) 107:187–241. 10.1016/B978-0-12-381300-8.00007-1 21034975 PMC7150010

[78] Duan S Thomas PG. Balancing immune protection and immune pathology by CD8(+) T-cell responses to influenza infection. Front Immunol. (2016) 7:25. 10.3389/fimmu.2016.00025 26904022 PMC4742794

[79] Chapman TJ Topham DJ. Identification of a unique population of tissue-memory CD4+ T cells in the airways after influenza infection that is dependent on the integrin VLA-1. J Immunol. (2010) 184:3841–9. 10.4049/jimmunol.0902281 20200271 PMC2843798

[80] Sakai S Kauffman KD Schenkel JM McBerry CC Mayer-Barber KD Masopust D. Cutting edge: control of Mycobacterium tuberculosis infection by a subset of lung parenchyma-homing CD4 T cells. J Immunol. (2014) 192:2965–9. 10.4049/jimmunol.1400019 24591367 PMC4010124

[81] Sallin MA Sakai S Kauffman KD Young HA Zhu J Barber DL. Th1 differentiation drives the accumulation of intravascular, non-protective CD4 T cells during tuberculosis. Cell Rep. (2017) 18:3091–104. 10.1016/j.celrep.2017.03.007 28355562 PMC5399512

[82] McKinstry KK Strutt TM Kuang Y Brown DM Sell S Dutton RW. Memory CD4+ T cells protect against influenza through multiple synergizing mechanisms. J Clin Invest. (2012) 122:2847–56. 10.1172/JCI63689 22820287 PMC3408751

[83] Quinones-Parra SM Clemens EB Wang Z Croom HA Kedzierski L McVernon J. A role of influenza virus exposure history in determining pandemic susceptibility and CD8+ T cell responses. J Virol. (2016) 90:6936–47. 10.1128/JVI.00349-16 27226365 PMC4944292

[84] Cadena AM Fortune SM Flynn JL. Heterogeneity in tuberculosis. Nat Rev Immunol. (2017) 17:691–702. 10.1038/nri.2017.69 28736436 PMC6247113

[85] Slight SR Rangel-Moreno J Gopal R Lin Y Fallert Junecko BA Mehra S. CXCR5(+) T helper cells mediate protective immunity against tuberculosis. J Clin Invest. (2013) 123:712–26. 10.1172/JCI65728 23281399 PMC3561804

[86] Lu LL Chung AW Rosebrock TR Ghebremichael M Yu WH Grace PS. A functional role for antibodies in tuberculosis. Cell (2016) 167:433–43 e14. 10.1016/j.cell.2016.08.072 27667685 PMC5526202

[87] Luthje K Kallies A Shimohakamada Y Belz GT Light A Tarlinton DM. The development and fate of follicular helper T cells defined by an IL-21 reporter mouse. Nat. Immunol. (2012) 13:491–8. 10.1038/ni.2261 22466669

[88] Galli SJ Tsai M Piliponsky AM. The development of allergic inflammation. Nature (2008) 454:445–54. 10.1038/nature07204 18650915 PMC3573758

[89] Seumois G Chavez L Gerasimova A Lienhard M Omran N Kalinke L. Epigenomic analysis of primary human T cells reveals enhancers associated with TH2 memory cell differentiation and asthma susceptibility. Nat Immunol. (2014) 15:777–88. 10.1038/ni.2937 24997565 PMC4140783

[90] Turner DL Goldklang M Cvetkovski F Paik D Trischler J Barahona J. Biased generation and *in situ* activation of lung tissue-resident memory CD4 T cells in the pathogenesis of allergic asthma. J Immunol. (2018) 200:1561–9. 10.4049/jimmunol.1700257 29343554 PMC5821590

[91] Shinoda K Hirahara K Iinuma T Ichikawa T Suzuki AS Sugaya K. Thy1+IL-7+ lymphatic endothelial cells in iBALT provide a survival niche for memory T-helper cells in allergic airway inflammation. Proc Natl Acad Sci USA. (2016) 113:E2842–51. 10.1073/pnas.1512600113 27140620 PMC4878506

[92] Hogg JC Chu F Utokaparch S Woods R Elliott WM Buzatu L. The nature of small-airway obstruction in chronic obstructive pulmonary disease. N Engl J Med. (2004) 350:2645–53. 10.1056/NEJMoa032158 15215480

[93] Morimoto Y Hirahara K Kiuchi M Wada T Ichikawa T Kanno T (2018). Amphiregulin-producing pathogenic memory T helper 2 cells instruct eosinophils to secrete osteopontin and facilitate airway fibrosis. Immunity 49:134–50 e6. 10.1016/j.immuni.2018.04.023 29958800

[94] Arpaia N Green JA Moltedo B Arvey A Hemmers S Yuan S. A distinct function of regulatory T cell*s* in tissue protection. Cell (2015) 162:1078–89. 10.1016/j.cell.2015.08.021 26317471 PMC4603556

[95] Wong MT Ong DE Lim FS Teng KW McGovern N Narayanan S. A high-dimensional atlas of human T cell diversity reveals tissue-specific trafficking and cytokine signatures. Immunity (2016) 45:442–56. 10.1016/j.immuni.2016.07.007 27521270

[96] Ganesan AP Clarke J Wood O Garrido-Martin EM Chee SJ Mellows T. Tissue-resident memory features are linked to the magnitude of cytotoxic T cell responses in human lung cancer. Nat Immunol. (2017) 18:940–50. 10.1038/ni.3775 28628092 PMC6036910

[97] Hombrink P Helbig C Backer RA Piet B Oja AE Stark R. Programs for the persistence, vigilance and control of human CD8(+) lung-resident memory T cells. Nat Immunol. (2016) 17:1467–78. 10.1038/ni.3589 27776108

[98] Maekawa Y Ishifune C Tsukumo S Hozumi K Yagita H Yasutomo K. Notch controls the survival of memory CD4+ T cells by regulating glucose uptake. Nat Med. (2015) 21:55–61. 10.1038/nm.3758 25501905

[99] Helbig C Gentek R Backer RA de Souza Y Derks IA Eldering E. Notch controls the magnitude of T helper cell responses by promoting cellular longevity. Proc Natl Acad Sci USA. (2012) 109:9041–6. 10.1073/pnas.1206044109 22615412 PMC3384214

[100] Steinert EM Schenkel JM Fraser KA Beura LK Manlove LS Igyarto BZ. Quantifying memory CD8 T cell*s* reveals regionalization of immunosurveillance. Cell (2015) 161:737–49. 10.1016/j.cell.2015.03.031 25957682 PMC4426972

[101] Goltsev Y Samusik N Kennedy-Darling J Bhate S Hale M Vazquez G. Deep profiling of mouse splenic architecture with CODEX multiplexed imaging. Cell (2018) 174:968–81. 10.1016/j.cell.2018.07.010 30078711 PMC6086938

[102] Strutt TM McKinstry KK Kuang Y Bradley LM Swain SL. Memory CD4+ T-cell-mediated protection depends on secondary effectors that are distinct from and superior to primary effectors. Proc Natl Acad Sci USA. (2012) 109:E2551–60. 10.1073/pnas.1205894109 22927425 PMC3458385

